# Risk factors for low birth weight in Botucatu city, SP state, Brazil: a study conducted in the public health system from 2004 to 2008

**DOI:** 10.1186/1756-0500-5-60

**Published:** 2012-01-23

**Authors:** Cátia Regina Branco da Fonseca, Maria Wany Louzada Strufaldi, Lídia Raquel de Carvalho, Rosana Fiorini Puccini

**Affiliations:** 1 Department of Pediatrics, Julio de Mesquita Filho São Paulo State University, Botucatu Medical School, Botucatu, SP, Brasil; 2 Department of Pediatrics, Federal University of São Paulo, São Paulo, SP, Brasil; 3 Department of Biostatistics, Julio de Mesquita Filho São Paulo State University, Institute of Biosciences, Botucatu, SP, Brasil

## Abstract

**Background:**

Low birth weight (LBW), defined as birth weight less than 2500 g, has a complex etiology and may be a result of premature interruption of pregnancy or intrauterine growth restriction. The objective of this study was to provide information on determinants of LBW and contribute to the understanding of the problem in Brazil.

**Methods:**

A case-control study was conducted in Botucatu city, SP state, Brazil. The study population consisted of 2 groups with 860 newborns in each group as follows: low weight newborns (LWNB) and a control group (weight ≥ 2500 g). Secondary data from 2004 to 2008 were collected using the Live Birth Certificate (LBC) and records from medical charts of pregnant women in Basic Health Units (BHU) and in the Public University Hospital (UH). Variables were as follows: maternal socio-demographic characteristics, pregnancy and birth conditions including quality of prenatal care according to 3 criteria. They were based on parameters established by the Ministry of Health (MH), one of them, the modified Kessner Index. The multivariable analysis by logistic regression was used to evaluate the association between variables and LBW.

**Results:**

According to the analysis, the factors associated with LBW were as follows: prematurity (OR = 56.98, 95% CI 29.52-109.95), twin pregnancy (OR = 20.00, 95% CI 6.25-100.00), maternal smoking (OR = 2.12, 95% CI 1.33-3.45), maternal malnourishment (OR = 2.30, 95% CI 1.08-5.00), maternal obesity (OR = 2.30, 95% IC 1.18-4.48), weight gain during pregnancy less than 5 kg (OR = 2.63, 95% CI 1.35-5.00) and weight gain during pregnancy more than 15 kg (OR = 2.26, 95% CI 1.16-4.41). Adequacy of prenatal care visits adjusted to gestational age was less frequent in the LBW group than in the control group (68.7% vs. 80.5%, x^2 ^*p *< 0.001). According to the modified Kessner Index, 64.4% of prenatal visits in the LWNB group were adequate.

**Conclusion:**

LWNB are a quite heterogeneous group of infants concerning their determinants and prevention actions against LBW and the follow-up of these infants have also been very complex. Therefore, improvement in the quality of care provided should be given priority through concrete actions for prevention of LBW.

## Background

Birth weight reflects gestational conditions and development in the fetal period. In 1975, the WHO defined LBW as birth weight less than 2,500 grams, and considered it as a consequence of premature interruption of pregnancy and/or intrauterine growth restriction [1]. Even nowadays, the etiology is complex, and it has remained as a public health problem in many countries and different regions of the world [2].

Birth weight is an important biological determinant of newborn (NB) survival in adverse conditions. It shows fetal exposition to risk factors such as maternal unfavorable socioeconomic conditions, smoking habit, malnutrition and diseases as well as lack of attention to prenatal care and delivery. In addition, it plays a critical role in estimating newborns at higher risk of death and diseases mainly in the neonatal period [3-6].

LBW has been the object of several studies because, in addition to its important role in infant mortality and morbidity, it poses risk on its growth and development. Studies in Brazil and Latin America have reported low weight as an important indirect cause of newborn deaths and its association with main causes of neonatal death, such as preterm birth, serious infections and perinatal asphyxia [7,8].

These newborns have schooling and health problems of different types which can impair their physical, cognitive and mental growth. These children are likely to develop nutritional problems during their childhood affecting their growth and development [9]. Studies developed in Brazil [10,11] on the correlation between obesity and overweight during adolescence with low birth weight, reported approximately 20% of this diagnosis in the study population, which led the authors to point out for some possible changes in nutritional patterns of these children.

Much research has been conducted aiming at investigating low birth weight, a public health issue, through discussion of either its etiology and population disparities, or its consequences and possible prevention [12-15].

Some studies point out as most frequent factors associated with LBW some maternal conditions such as low education level, the very young or very old ages, inappropriate gestational weight gain, previous malnutrition and smoking habits [14,16] and some newborn conditions such as prematurity, prenatal follow-up with few consults or lack of them [6].

Therefore, determinants of low birth weight have been controversial in the literature, which explains the difficulties finding factors that are always associated with this event, as different social realities can be distinguished in places where the studies are conducted.

The Brazilian overview of the last years reveals an apparently paradoxical behavior, as LBW rates are higher in more developed regions. In the 90 s, a comparative study conducted in a less developed region of the northeast and in a well developed region of the south revealed LBW ratio of 9.71%: 8.08%, respectively. By 2004, this ratio was inverted, 7.57% in the northeast and 8.52% in the south [17]. In an effort to understand this paradox in official data, some studies were conducted and no significant reduction was found in LBW prevalence also in other cities in the country.

This regional difference has been currently attributed to the availability of prenatal and perinatal care rather than to social condition. The latter was the main cause of low birth weight in the last decade in less developed regions in the country [18].

Understanding this local and national realities as well as LWB determinants to both prevent birth and death of these newborns and comprehend possible alterations in their development is crucial for planning actions to enhance the Public Health System, and as a consequence the survival and prognosis of these infants [19].

The Prenatal and Birth Humanization Program (PHPN) was set up in 2000 to offer strategic actions to improve care quality for the pregnant woman and her fetus, and it brought in its core the discussion about prenatal practices and their conceptual basis according to patterns used worldwide. The major strategy of PHPN is to ensure improvement in the access, coverage and quality of prenatal follow-up, delivery and puerperal care for the pregnant woman and newborn, in the perspective of citizenship rights, following specific and well defined actions which must be closely monitored during prenatal care [20].

According to the National Survey on Health and Demography (PNDS) in 2006, Botucatu and most cities in the southeastern region, especially in São Paulo state, were found to have prenatal coverage of 6 or more consults for approximately 80% of pregnant women [21].

Despite good coverage in prenatal visits, a stability in the rates of low birth weight and neonatal mortality could be found in the period of the study in Botucatu [17,22]. These rates were higher than those reported in São Paulo state. The current study arose from the need of analyzing the determinants of low birth weight in the city, considering that, despite the awareness of these rates by local health authorities, no study using this specific analysis had been performed up to the time of the study. Therefore, this study aimed at identifying LBW-related factors in infants in Botucatu contributing to better understanding of this problem in the approach of the public health system in Brazil, and triggering the discussion of the importance of supportive public policies and health actions for women and infants.

## Methods

A case-control study with secondary data collection was conducted in the mid-southern region of São Paulo state, Botucatu city, southeastern Brazil. The city has a predominantly urban population (96%) of 127,370 inhabitants [23]. By 2005, with a Human Development Index (HDI) of 0.822, the city was ranked 56th among 645 cities in the state, and 201th among the cities in the country. In addition, the HDI-Education and Income components in that year were 0.774 and 0.783, respectively, which were considered medium values [24].

According to data of the State System Foundation for Data Analysis (SEADE) based on the Information System of Live Births (SINASC) in the years of 2004, 2005, 2006, 2007 and 2008, the number of live births were 1667, 1702,1670, 1675 and 1728 respectively, amounting 8442 live newborns. Data of DATASUS for these same years showed low birth weight rates per year of 9.6%, 9.1%, 10.5%, 12.2% e 9.3%, respectively, amounting 860 children with low birth weight [17,22].

All live births whose mothers lived in Botucatu at the time of delivery in the period from 2004 to 2008 were included in the study. The newborns of the Case and Control groups were from this population. The Case group consisted of all low weight newborns (with less than 2500 gr) amounting 860 infants, and the Control group consisted of a randomized sample of 860 live newborns weighing ≥ 2500 gr. For each year considered in the study, the same number of NB were selected for the Case group (LBW) and Control group (non LBW).

**Figure 1:**
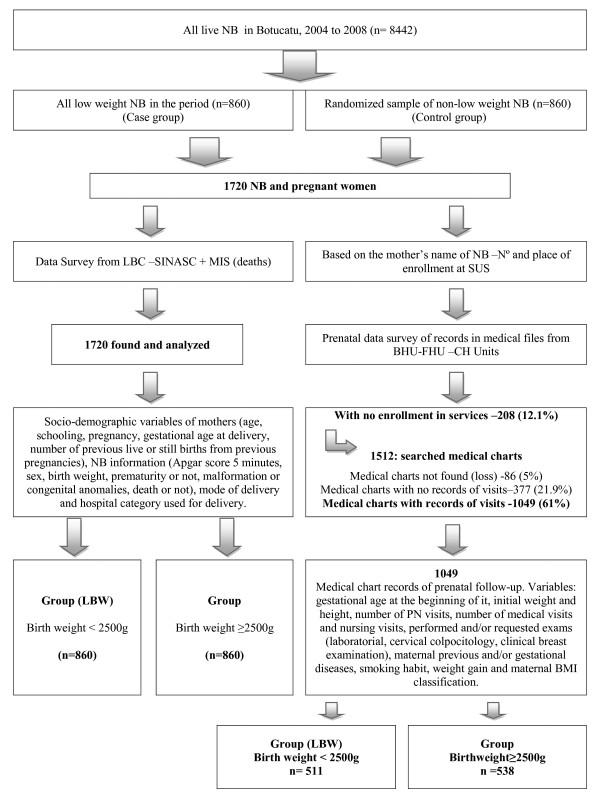
**Flowchart of the study population, data source, information assessed and study groups**.

Figure 1 shows the variables used per categories and the secondary sources of data. The secondary sources were as follows: Live Birth Certificate through SINASC, and records on medical charts of pregnant women concerning prenatal care and maternal conditions during pregnancy in Health Units registered in the Unified Health System (SUS) such as Basic Health Units (BHU) and the Public University Hospital (UH). The focus of this study was the evaluation of SUS users. Therefore, collection of records in medical charts was made in these health units. Information about whether or not there had been deaths in the first year of age was obtained from the Mortality Information System [17] (Figure 1). A total of 1720 protocols for all newborns were fulfilled, in which 1049 charts contained records of pregnant women with prenatal follow-up by the Unified Health System. The procedures which should be in the charts, according to the Humanization Program of Prenatal care and Birth (PHPN) [20], but were not recorded, were considered as not performed.

For analyses of adequacy and prenatal care quality in HBUs, three criteria were considered: 1. *Number of prenatal care visits at gestational age *(Table 1); 2. *Modified Kessner Index *- adequate number of prenatal care visits by gestational age and beginning of prenatal follow-up. The modified Kessner Index was considered for the Brazilian proposal in the PHPN [20,25,26] and the score was assigned as follows: adequate-when 100% of expected visits were held and the beginning of prenatal follow-up was held up to 17 weeks (fourth month); inadequate-when the beginning of prenatal care was after that period or number of prenatal visits were fewer than 100% of the expected ones, and 3. *Adequacy of prenatal care *- It considered the number of prenatal visits by gestational age added to the procedures recommended routinely in Brazil, such as complementary exams (cervical colpocitology and laboratorial exams); clinical breast examination recorded in any medical visit during the gestational period; at least one ultrasound and beginning of follow-up until the 17th week. Prenatal care was considered adequate when having this range of events, and inadequate, when other different situations were present.

**Table 1:** Proposal of adequacy of prenatal care visits by gestational age

Gestation (weeks)	Number of prenatal care visits^1^
22 or less	1-3

23-27	4 or more

28-31	5 or more

32-36	6 or more

37 or more	7 or more

^1 ^Adequacy from PHPN and Kessner Index [20,25,26]

### Ethics

Following the ethical principles established in the Helsinki Declaration, this study was evaluated and approved by the Research Ethics Committee (REC) of Botucatu Medical School/UNESP (3372/2009), and by the REC of UNIFESP (0280/2010) [27].

### Data Analysis

Chi-square Test was used to evaluate the association among variables. The significance level was 5% (alpha = 0.05) for rejection of the null hypothesis [28]. In the multivariable analysis, the logistic regression model was used by the Statistical Package for the Social Sciences Program (SPSS) [29] to investigate "low birth weight" as a dependent variable in relation to other variables of interest. To identify variables with higher association with Low Birth Weight multivariable analyses were done, firstly using a "non adjusted" model which considered the variables singly, and related them to low birth weight, and secondly using an "adjusted model", which considered all factors involved in one only model simultaneously. For multivariable analyses, p-value, calculated by Wald test, was considered significant for p ≤ 0.05 [29]. To determine the effect of each variable on LBW, *Odds Ratio *was calculated based on 95% confidence interval in the adjusted and unadjusted model.

## Results

Using data of SEADE Foundation and DATASUS, the prevalence of low birth weight in the period of the study was 10.2% considering all live newborns in the period in the denominator (8442) [17,22].

Data on 1720 newborns included in the study were obtained in LBC and information on 1049 pregnant women (61%) from medical charts in BHU and UH, in which 48.7% (511) were in the LWNB group and 51.3% (538) in the NB group weighing ≥ 2500 g, as shown in the flowchart (Figure 1). The difference was not statistically significant and no variable included in the analysis had more than 20% of missing data.

Distribution of maternal gestational and delivery characteristics as well as those of newborns according to the birth weight group is shown in Additional file 1: Table S1. In this analysis, the group of LBWNB had higher percentage of mothers less than 12 years of schooling (88.4%), being 32.5% of them with less than 8 years of schooling; higher frequency of adolescent mothers (22.6%), being 1.4% less than 15 years old, 63.8% of preterm pregnancy and 50.2% of primigravid women. A total of 36.2% term pregnancies led to LWNB and this percentage of NB could be attributed to restriction of intrauterine growth. LBW was more frequent in twin pregnancies (16.4% vs. 0.7%, *p *< 0.001), and these NB had higher incidence of deaths in the first year of life (6.3% vs. 0.0%, *p *< 0.001) and higher rate of serious and moderate neonatal depression (7.8% vs. 1.7%, *p *< 0.001).

For LWNB, the number of cesarean deliveries, more often in UH, was higher than that of vaginal deliveries (55.1% vs. 44.6%, *p *< 0.001) (Additional file 1: Table S1).

Additional file 2: Table S2 shows the analysis of maternal gestational variables associated with LBW, which were as follows: smoking during gestation (23.3% vs. 14.3%, *p *< 0.001), diagnosis of maternal malnourishment (7.3% vs. 3.5%, *p *= 0.041), weight gain lower than the expected one (19.6% vs. 13.3%, *p *< 0.001), maternal history of systemic arterial hypertension (6.5% vs. 1.7%, *p *< 0.001) and diseases during the current pregnancy (vulvovaginitis, gestational hypertension and preeclampsia, all of them with higher incidence in the LBWNB group, *p *< 0.001).

Additional file 3: Table S3 shows that pregnancies which led to LWNB had fewer prenatal visits (*p *< 0.001) and medical visits (*p *< 0.001) compared to those in the control group. Also, no nursing visits were accomplished during most of these pregnancies (*p *< 0.001). A high number of ultrasound exams were performed in both groups and the small number of subjects who did not undergo this exam drew our attention (10.9% vs. 9.0%, *p *< 0.001). Among the routine exams recommended by the Ministry of Health, breast clinical examination had the lowest frequency in prenatal visits (62.0% vs. 60.8%, *p *= 0.274).

The analysis of adequacy and quality of prenatal care through the 3 proposed indicators in Additional file 3: Table S3, showed that a significant difference between groups (*p *< 0.001) was observed regarding only to the number of adequate prenatal care visits by gestational age, with a higher frequency of inadequate care related to LBW (31.3%) in relation to the control group (19.3%).

Additional file 4: Table S4 shows adjusted and non adjusted results of multivariable analysis (logistic regression model). According to them, gestational age less than 37 weeks (OR = 56.98, 95% CI 29.52-109.95, *p *< 0.001), twin birth (OR = 20.00, 95% CI 6.25-100.00, *p *< 0.001), maternal malnourishment (OR = 2.30, 95% CI 1.08-5.00, *p *= 0.029) or obesity (OR = 2.30, 95% CI 1.18-4.48, *p *= 0.015), inadequate weight gain being less than 5 kg (OR = 2.63, 95% CI 1.35-5.00, *p *= 0.004) or more than 15 kg (OR = 2.26, 95% CI 1.16-4.41, *p *= 0.017) and also smoking during pregnancy (OR = 2.12, 95% CI 1.33-3.45, *p *= 0.002) were associated with LBW.

The multivariable analysis by logistic regression including the 3 criteria for evaluation of prenatal care quality was applied and no associations with low birth weight were found considering the adjusted and non adjusted models. No influence of prenatal care quality on the association with other factors and LBW was observed.

## Discussion

The study aimed at identifying factors associated with low birth weight over 5 years in a mid- sized city in São Paulo state interior region, Brazil, and expanding the discussion about this theme in the literature. For that purpose, a control group was set up to compare variables and search different data sources for a joint discussion about maternal socio-demographic factors, those related to delivery and to the newborn and in a highly comprehensive manner, the prenatal care provided for these pregnant women.

Data and outcomes from this study revealed the reality of the city in the study years concerning characterization of low birth weight newborns, their conditions of prenatal follow-up and birth as well as maternal health and socio-demographic situation, considering that the study analyzed the world of the LWNB and used a sample, for technical feasibility, of non-low weight NB as a control group so that the comparison between them was made.

Low birth weight rate for the city of Botucatu has been higher, except for the year 2008, than the rates for São Paulo state [17], the South (8.7%) and the Southeast region (9.1%) and it has been higher than the rates found for less developed regions in the country (North 7.0% and Northeast 7.4%) [18].

Currently, reduction in these regional disparities in Brazil has been attributed to prematurity rates, which have been increasing in the country as a whole, and kept low rates of birth weight. Simultaneously, in the last decade, a reduction in term children with low birth weight (intrauterine growth restriction) has been observed. Therefore, low birth weight has been associated rather to conditions of access to health care and services than to socio economic conditions [30].

In this study, factors associated with low weight were prematurity, twin births, maternal malnourishment and obesity, inadequate weight gain less than 5 kilos or more than 15 kilos and maternal smoking. Considering the reality in which the study was conducted, i.e., in a city with good socio-economic conditions, a good score in the HDI classification [24], high level of maternal schooling and low rate of illiteracy, these variables and maternal age were not associated with higher risk of LBW. These findings are in agreement with those reported in some studies conducted in Brazil and in other countries [14,31,32].

Therefore, no conclusions can be reached on the association between socio economic and demographic conditions and LBW. This finding is a consequence of different socio economic realities in the study regions, since they are interconnected factors related to life conditions of the population such as, housing, income, basic sanitation, level of knowledge and access to health services which directly influence favorable or unfavorable outcomes in the health-disease process, including pregnancy and birth conditions.

A stronger association of LBW with prematurity and not with intrauterine growth restriction is pointed out in this study. Also, twin pregnancies are often associated with shorter period of gestation. The association has already been described in Brazil and worldwide, and currently it has been considered the main factor for keeping low birth weight rates in Brazil [2,30,33].

The importance of maternal smoking during pregnancy and its relationship with low birth weight is reported in the literature and also confirmed in this study as an important predictor of LBW. This finding was one of the first consequences of this habit, and it is probably related to intrauterine hypoxia leading to fetal malnutrition [34,35]. Some studies in United States and Canada have reported that smoking reduction during pregnancy may decrease the LBW incidence in developed countries like USA and Canada [36,37].

The infant mortality rate of the Low Birth Weight newborn group in our study shows the local reality and represents 60 deaths per 1000 births. No deaths were reported in the control group. However, data analysis from the SEADE Foundation [22], which reports IM rates and number of deaths per year in the city, revealed a rate of 0.7% for this population, that is, 7 infant deaths per 1000 births. The low value obtained in this study for the Apgar score at 5 minutes, mostly in the LBW group, could be associated with the higher frequency of infant deaths in this group of NB. Neonatal depression maintained at 5 minutes in Apgar score represents maintenance of adverse conditions with consequences for the prognosis of these NB who have already been compromised by inadequate birth weight and short gestational age. Also, it contributes to higher rates of neonatal mortality [38].

Maternal nutritional status and weight gain during pregnancy have been addressed in many studies. According to Melo et al. [39] "not only to the growing prevalence of their disorders but also to their important role in gestational outcomes, such as fetal growth and birth weight [...]". A reduction in calorie intake during pregnancy because of distinct reasons is the cause of low birth weight in NB, and the literature on this issue is historical and ancient. Low weight gain causes serious harm and may account for higher morbimortality rates of these NB. The most used concept of adequacy, even in this study, has been the one which considers adequate weight gain as 10 to 15 kg [40-42].

Many researchers have evaluated the association between previous maternal diseases and gestational diseases and low weight of newborns [35,43]. Some studies reported a reliable association among them. However, other studies in the literature and also the present study found a different association. In this study, an initial positive association between LBW and some diseases (systemic and gestational hypertension and gestational diseases) was observed, although it was not verified in the multivariable analysis.

For the city of Botucatu, which has a diversified public health care both in Basic Health Units (BHU) and in the Public University Hospital, as well as good level of maternal schooling, the situation mentioned above is an expected reality considering that pregnant women awareness and the access to health services ensure them an adequate disease control.

The Public Health System in Brazil follows organizational principles, and the hierarchical health care is one of them. [44] The referral to services which require advanced technology and more expertise must be done in cases in which the health primary attention has identified them as at higher risk. Based on our findings, it could be observed that this organizational system of SUS has appropriately led to a higher number of LWNB deliveries in the public University Hospital. This demand for services with high level of technology rises the cost of health care in the Brazilian Public Health Service, which is a universal system funded by taxes and specific contributions [45]. In addition, higher occurrence of cesarean deliveries in this group of NB as compared to that in the adequate weight group was found, which justifies both situations, place and type of delivery, since these NB and their pregnancies are at higher risk as a consequence of maternal or fetal complications at birth. The place of delivery could be justified as a search for better perinatal outcomes, since University Hospitals, with characteristics of tertiary care, offer adequate support for neonatal care (neonatal ICU) usually required by these NB [46].

Data from Brazil show that there have been high coverage rates for prenatal follow-ups with 80.7% of women having 5 or more prenatal consults between 2006 and 2007, and 83.6% of them, starting the prenatal follow-up within the first trimester of gestation. Therefore, the present concern relies on quality evaluation of the care provided.

A high coverage rate for prenatal care was also observed in our study, which shows the appropriate understanding of the need for follow-ups by the pregnant women. Moreover, it shows the wide offer of this service in the health care system.

Prenatal care is one of the most ancient care practice in the Brazilian Health System, and an optimal condition would be the one in which medical care could be offered to women since the very beginning of the pregnancy diagnosis. For that purpose, health services must have skilled professionals to appropriately welcome and build close ties with the users. Therefore, the main determinant of low quality care provided by the SUS network has been the limited human resources. However, this limitation is qualitative rather than quantitative [47].

Care of pregnant women must include minimal procedures and necessary exams to reduce maternal and fetal morbidity and mortality. This care would be through prevention and early diagnosis of alterations as intrauterine growth restriction and maternal infections which can be vertically transmitted to the offspring [42]. A good prenatal follow-up ensures a protective effect for the pregnant woman and fetus through better nutritional control, access to resources for reducing smoking, and early diagnosis and adequate treatment of infections and diseases which could prevent low birth weight in newborns.

According to some studies, neonatal mortality, prevalence of low birth weight and prematurity in Brazil are related to lack of access to routine and basic procedures concerning pregnancy assistance [13].

One of the objectives of this study was to evaluate prenatal care and its relationship with low birth weight. The modified Kessner Index, Adequacy Prenatal Care Utilization Index and Kotelchuck Index are combined measurements more frequently used to a better study of quality of prenatal care utilization [25,26]. The criterion used to evaluate quality of prenatal follow-up is essential, and the more complex and comprehensive it is, the better the identification of the reality in which the study took place, enabling data collection for discussion about the care delivered. This study revealed a reduction in the adequacy indexes for both groups according to the increase in coverage of the indicator used.

The increase in inadequacy as a result of the modified Kessner Index utilization, as compared to the analysis which considered only the number of visits at the gestational age, shows that the late start of prenatal follow-up was the contributing factor in this reduction in adequacy.

Therefore, when variables are associated for a judicious evaluation of prenatal quality, different results are found compared to those from a more partial or fragmented analysis of care, even when there is no association with LBW as in the present study.

Attention to adequate prenatal care should not be limited to prevention of LBW, as this service ensures advising for pregnant women concerning their health, and adoption of healthy life habits for themselves and their infants. Moreover, it identifies risk situations for pregnancy and delivery, interventions are timely applied and unfavorable outcomes for the mother or newborn are avoided. Also, mothers may be enrolled in programs of health care attention to the woman and infant for later follow-ups.

Data from this study and from literature show that the risks for LWNB are fully established and they may vary according to the social and economic reality in which the study was conducted. However, this problem must be acknowledged and faced by the Health Public System. Although the widespread knowledge of the risk for low birth weight in Sao Paulo state and Brazil, the specific evaluation of the prenatal follow-up and control of these factors have not been widely and uniformly performed in Brazilian cities. The adequate identification of LBW associated factors must create effective and preventive strategies, and the birth of LWNB must give rise to a necessity of redesigning intra and extra hospital actions to better welcome and follow the triad of the NB/mother/family, reducing risks and promoting better growth and development of this infant.

Few limitations were found in this study, as for the object of the study (LBW), all cases in the study period were included. The proposal of identifying prenatal care associated factors and considering births within the Public Health System led to the incorporation of most pregnancies and births occurred in the city. However, because of the great population diversity, the study of prenatal care in private or managed health care settings could bring other contributions to the discussion of this issue.

## Conclusion

Most factors associated with LWNB in this study would be avoidable if health services were structured to timely recruit pregnant women at the very beginning of their pregnancy, and provide prenatal follow-ups which would include the pregnant women welcome and control of the absentees. This process would enable early detection of risks and promotion of actions through interdisciplinary and inter sectoral approaches aiming at changing habits, diagnosing diseases, providing better nutritional conditions and monitoring gestational diseases. Based on epidemiological characteristics, LBW newborn must be considered rather a sentinel event than a risk indicator. This sentinel event would have a potentially avoidable occurrence: the infant death. A qualitative analysis with individual information examined on a case by case basis could provide further clarification of this Public Health issue which has not been altered in the last decade in Brazil and worldwide.

The study shows that LWNB is a very heterogeneous group of children concerning its determinants, and therefore, actions to prevent LBW and follow-up of these children have been a challenge for the Public Health System. This study on risk factors associated with low birth weight in the city of Botucatu as well as others conducted in the country have as their main objective to improve comprehension of this problem so that concrete actions towards it are incorporated. However, lowering these rates of LWNB is not an easy task because of its multifactorial and complex etiology and differentiated display according to the city or region in the study. Cities have different socio-demographic characteristics and health service structure as reported in our study and in the literature.

While municipal health programs for pregnancy, delivery and newborn care have been improved as priorities of local and national public policy, structuring health services regarding an adequate follow-up of these children after birth; increasing the number of openings and improving resources in Neonatal Intensive Care Units; providing ward services for an adequate follow-up and prevention of sequelae in these children should also be a political priority.

Therefore, the contribution of this study to the area is to show that risk factors for low birth in the city may be overcome. Ultimately, the challenge to be faced by health authorities has currently been political, so that the right to health care has to be ensured to all pregnant women and newborns through improved and already well defined actions towards health care.

## Competing interests

The authors declare that they have no competing interests.

## Authors' contributions

All authors have made substantial contributions to the study and endorsed data and conclusions. CRBF identified the research question, conducted the analyses and wrote the first draft of the article. LRC contributed to the analysis and assisted in editing of the article. RFP, MWLS contributed to identification of the research question, interpretation of findings and writing of the article. All authors have read and approved the final version of the manuscript.

## Supplementary Material

Additional file 1 **Table S1**. Distribution of socio-economic characteristics, newborn and gestational conditions, hospital category and mode of delivery. Click here for file

Additional file 2 **Table S2**. Maternal habits, diseases and conditions during gestation and distribution in the study groups. Click here for file

Additional file 3 **Table S3**. Characterization of prenatal follow-up and adequacy indexes of care in the study groups. Click here for file

Additional file 4 **Table S4**. Factors associated with low birth weight and multivariable analysis by logistic regression. Click here for file
