# Supplementary material for: Risk factors for low birth weight in Botucatu city, SP state, Brazil: a study conducted in the public health system from 2004 to 2008
Source: BMC Res Notes. 2012 Jan 23;5:60. doi: 10.1186/1756-0500-5-60 (PMC3285524; doi:10.1186/1756-0500-5-60)
Supplement: Additional file 1 — Table S1. Distribution of socio-economic characteristics, newborn and gestational conditions, hospital category and mode of delivery. [file 1756-0500-5-60-S1.PDF]

Table 2. Distribution of socio-economic characteristics, newborn and gestational conditions, hospital category and mode of delivery.

Variables	Groups				*p*
	Birth weight <2500g (LBW)		Birth weight ≥2500g		
	n=860	%	n=860	%	
**Maternal schooling (years)**					**0.013**
0	5	0.6	5	0.6	
1 - 3	30	3.5	27	3.1	
4 - 7	244	28.4	224	26.0	
8 - 11	481	55.9	452	52.6	
≥12	100	11.6	152	17.7	
**Maternal age (years)**					**< 0.001**
<15	12	1.4	4	0.5	
15 - 19	182	21.2	135	15.7	
20 - 34	577	67.1	648	75.4	
≥35	89	10.3	73	8.4	
**Gestational age (weeks)**					**<0.001**
< 22	2	0.2	0	-	
22 - 27	46	5.4	0	-	
28 - 31	100	11.6	0	-	
32 - 36	401	46.6	30	3.5	
37 - 41	311	36.2	824	95.8	
≥42	0	-	6	0.7	
**Preterm birth**					**<0.001**
Yes	549	63.8	36	4.2	
No	311	36.2	824	95.8	
**Parity (Previous live birth)**					**0.017**
0	432	50.2	379	44.1	
1	195	22.7	268	31.2	
2	125	14.5	113	13.1	
≥3	108	12.6	100	11.6	
**Previous stillbirth/abortion**					0.580
0	836	97.2	837	97.3	
1	24	2.8	22	2.6	
≥2	0	-	1	0.1	
**Type of pregnancy**					**< 0.001**
Singleton	719	83.6	854	99.3	
Twin	141	16.4	6	0.7	
**Newborn sex**					0.455
Female	446	51.9	431	50.1	
Male	414	48.1	429	49.9	
**Newborn with congenital malformation**					0.069
Yes	17	2.0	8	0.9	
No	843	98.0	852	99.0	
**Death of newborn (≤ one year)**					**< 0.001**
Yes	54	6.3	0	-	
No	806	93.7	860	100.0	
**Apgar score at 5 minute[1]**					**< 0.001**
0 - 3	21	2.5	03	0.3	
4 - 6	37	4.3	12	1.4	
7 - 10	802	93.2	845	98.3	
**Hospital category**					**< 0.001**
Public University	509	59.2	239	27.8	
Charitable - a joint venture with the Public Health System	214	24.9	449	52.3	
Private	135	15.7	169	19.6	
Non-hospital	2	0.2	3	0.3	
**Mode of delivery**					**< 0.001**
Vaginal	386	44.9	476	55.4	
Cesarean	474	55.1	384	44.6	

Source: Live Birth Reports. [1] Missing 0.1%.
