# Supplementary material for: Risk factors for low birth weight in Botucatu city, SP state, Brazil: a study conducted in the public health system from 2004 to 2008
Source: BMC Res Notes. 2012 Jan 23;5:60. doi: 10.1186/1756-0500-5-60 (PMC3285524; doi:10.1186/1756-0500-5-60)
Supplement: Additional file 2 — Table S2. Maternal habits, diseases and conditions during gestation and distribution in the study groups. [file 1756-0500-5-60-S2.PDF]

Table 3. Maternal habits, diseases and conditions during gestation and distribution in the study groups.

**Variables**	**Group**				***p***
	**Birth weight < 2500g (LBW)**		**Birth weight ≥ 2500g**		
	**n= 511**	**%**	**n= 538**	**%**	
**Maternal smoking**					**< 0.001**
No	335	65.5	386	71.8	
Yes	119	23.3	77	14.3	
Missing	57	11.2	75	13.9	
**Weight gain during pregnancy (kg)**					**< 0.001**
≤ 5	100	19.6	70	13.0	
5.1 - 10	194	37.9	159	29.6	
10.1 - 15	103	20.2	166	30.9	
>15	42	8.2	117	21.7	
Missing	72	14.1	26	4.8	
**Classification by maternal BMI (kg/m²)**					**0.041**
Malnourished	37	7.3	19	3.5	
Eutrophic	283	55.4	276	51.3	
Overweight	47	9.2	60	11.2	
Obese	88	17.2	100	18.6	
Missing	56	10.9	83	15.4	
**Previous maternal diseases**					0.282
No	341	66.8	382	71.0	
Yes	141	27.6	132	24.5	
Missing	29	5.6	24	4.5	
**Most common diagnoses [1]**					
Vulvovaginitis	15	2.9	16	3.0	0.979
Systemic Hypertension	33	6.5	9	1.7	**< 0.001**
Maternal cardiopaty	7	1.4	5	0.9	0.498
Other	113	25.2	119	22.1	0.952
**Diseases during pregnancy[1]**					**< 0.001**
No	211	41.3	300	55.8	
Yes	270	52.9	210	39.0	
Missing	30	5.8	28	5.2	
**Most common diagnoses**					
Gestational vulvovaginitis	139	27.2	97	18.0	**< 0.001**
Gestational UTI[2]	80	15.7	71	13.2	0.246
Gestational hypertension	66	12.9	20	3.7	**< 0.001**
Pre-eclampsia	39	7.6	5	0.9	**< 0.001**
Other	63	12.3	80	14.8	0.465

Source: Records of pregnant women at the Primary Health Care Units and Botucatu University Hospital

[1] There were pregnant women with more than one disease; [2] UTI = Urinary tract infection.
