# Supplementary material for: Risk factors for low birth weight in Botucatu city, SP state, Brazil: a study conducted in the public health system from 2004 to 2008
Source: BMC Res Notes. 2012 Jan 23;5:60. doi: 10.1186/1756-0500-5-60 (PMC3285524; doi:10.1186/1756-0500-5-60)
Supplement: Additional file 3 — Table S3. Characterization of prenatal follow-up and adequacy indexes of care in the study groups. [file 1756-0500-5-60-S3.PDF]

Table 4. Characterization of prenatal follow-up and adequacy indexes of care in the study groups.

Variables	Group Birth weight <2500g (LBW)		Birth weight ≥2500g		p^4
	n= 511	%	n= 538	%	
**Number of Prenatal care visits**					**< 0.001**
1 - 3	71	13.8	28	5.2	
4 - 6	137	26.8	77	14.3	
≥ 7	303	59.4	433	80.5	
**Number of medical visits**					**< 0.001**
0	24	4.7	24	4.5	
1 - 3	130	25.5	105	19.5	
4 - 6	172	33.6	132	24.5	
≥ 7	180	35.2	275	51.1	
Missing	05	1.0	02	0.4	
**Number of nurse visits**					**<0.001**
0	211	41.3	188	34.9	
1 - 3	140	27.4	120	22.3	
4 - 6	83	16.2	91	16.9	
≥ 7	63	12.3	137	25.5	
Missing	14	2.7	02	0.4	
**No prenatal care**	17	1.0	4	0.2	**< 0.001**
**Examinations performed**					
Laboratory[1]	470	91.9	510	94.8	0.138
Cervical citology[1]	401	78.5	430	79.9	0.544
Breast clinical exam[2]	317	62.0	327	60.8	0.274
**Ultrasound**					**< 0.001**
0	56	10.9	49	9.0	
1 - 3	381	74.7	461	85.6	
≥ 4	73	14.4	29	5.4	
**Adequacy of prenatal care visits by gestational age**					**<0.001**
Adequate	351	68.7	433	80.5	
Inadequate	160	31.3	105	19.5	
**Adequacy of prenatal care (adapted Kessner Index)[3]**					0.210
Adequate	299	64.4	330	61.3	
Inadequate	165	35.6	208	38.7	
**Adequacy of prenatal care (exams and adapted Kessner)[3]**					0.110
Adequate	204	44.0	217	40.3	
Inadequate	260	56.0	321	59.7	

Source: Records of pregnant women at the Primary Health Care Units and Botucatu University Hospital.

[1] Missing 1 (0.2%) birth weight group ≥2500g; [2] Missing 6 (1.1%), birth weight group ≥2500g; [3] Missing 47(9.2) LBW group; [4] Chi-Square Test.
