# Supplementary material for: Risk factors for low birth weight in Botucatu city, SP state, Brazil: a study conducted in the public health system from 2004 to 2008
Source: BMC Res Notes. 2012 Jan 23;5:60. doi: 10.1186/1756-0500-5-60 (PMC3285524; doi:10.1186/1756-0500-5-60)
Supplement: Additional file 4 — Table S4. Factors associated with low birth weight and multivariable analysis by logistic regression. [file 1756-0500-5-60-S4.PDF]

Table 5. Factors associated with low birth weight and multivariable analysis by logistic regression.

Variables	Non-adjusted[2]			Adjusted[2]		
	OR[1]	**[95% CI][3]**	***p*[4]**	***OR[1]***	**[95% CI]**	***p*[4]**
**Maternal schooling (years)**			**0.018**			0.102
< 8	5.18	2.97-12.01		0.44	0.16-1.18	
8 - 12	2.34	1.87-3.17		0.53	0.20-1.38	
>12	1.00	Reference		1.00	Reference	
**Gestational age (weeks)**			**< 0.001**			**< 0.001**
≥ 37	1.00	Reference		1.00	Reference	
< 37	1.03	1.02-1.04		56.98	29.52-109.95	
**Type of pregnancy**			**< 0.001**			**< 0.001**
Singleton	1.00	Reference		1.00	Reference	
Twin	2.89	1.94-3.85		20.00	6.25-100.00	
**Classification by maternal BMI[2]**			**< 0.001**			**0.015**
Malnourished	1.45	1.21-2.07		2.30	1.08-5.00	
Eutrophic	1.00	Reference		1.00	Reference	
Overweight	1.17	1.06-1.51		1.66	0.96-2.87	
Obese	2.09	1.42-4.74		2.30	1.18-4.48	
**Weight gain during pregnancy**			**< 0.001**			**0.004**
≤ 5Kg	1.13	1.05-1.35		2.63	1.35-5.00	
5.1 - 10 Kg	1.54	1.27- 2.23		1.61	0.99-2.63	
10.1 - 15 Kg	1.00	Reference		1.00	Reference	
> 15 Kg	7.64	1.16- 1.64		2.26	1.16-4.41	
**Maternal smoking**			**0.007**			**0.002**
Yes	7.64	3.68-23.88		2.12	1.33-3.45	
No	1.00	Reference		1.00	Reference	

Source: Live Birth Reports and records of pregnant women at the Primary Health Care Units and Botucatu University Hospital.

[1] OR = odds ratio*;* [2] Factors with p > 0.05 in association with LBW in multivariable analysis were not included in the non-adjusted and adjusted model; [3] 95%CI = confidence interval; [4] p values were derived using the Wald test.
